# MyoD- and nerve-dependent maintenance of *MyoD *expression in mature muscle fibres acts through the DRR/PRR element

**DOI:** 10.1186/1471-213X-8-5

**Published:** 2008-01-23

**Authors:** Sophie B Chargé, Andrew S Brack, Stéphanie A Bayol, Simon M Hughes

**Affiliations:** 1 Randall Division for Cell and Molecular Biophysics and the MRC Centre for Developmental Neurobiology, New Hunt's House, Guy's Campus, King's College London, London, UK; 2 Stem Cell Network, 451 Smyth Road, Room 3105, Ottawa, Ontario K1H 8M5, Canada; 3 Department of Neurology and Neurological Sciences, Stanford University School of Medicine, Stanford, CA, USA; 4 Royal Veterinary College, London NW1 0TU, UK

## Abstract

**Background:**

MyoD is a transcription factor implicated in the regulation of adult muscle gene expression. Distinguishing the expression of *MyoD *in satellite myoblasts and muscle fibres has proved difficult *in vivo *leading to controversy over the significance of *MyoD *expression within adult innervated muscle fibres. Here we employ the *MD6.0-lacZ *transgenic mouse, in which the 6 kb proximal enhancer/promoter (DRR/PRR) of *MyoD *drives *lacZ*, to show that MyoD is present and transcriptionally active in many adult muscle fibres.

**Results:**

In culture, *MD6.0-lacZ *expresses in myotubes but not myogenic cells, unlike endogenous *MyoD*. Reporter expression *in vivo *is in muscle fibre nuclei and is reduced in *MyoD *null mice. The *MD6.0-lacZ *reporter is down-regulated both in adult muscle fibres by denervation or muscle disuse and in cultured myotubes by inhibition of activity. Activity induces and represses *MyoD *through the DRR and PRR, respectively. During the postnatal period, accumulation of β-galactosidase correlates with maturation of innervation. Strikingly, endogenous *MyoD *expression is up-regulated in fibres by complete denervation, arguing for a separate activity-dependent suppression of *MyoD *requiring regulatory elements outside the DRR/PRR.

**Conclusion:**

The data show that *MyoD *regulation is more complex than previously supposed. Two factors, MyoD protein itself and fibre activity are required for essentially all expression of the 6 kb proximal enhancer/promoter (DRR/PRR) of *MyoD *in adult fibres. We propose that modulation of MyoD positive feedback by electrical activity determines the set point of *MyoD *expression in innervated fibres through the DRR/PRR element.

## Background

Myogenic regulatory transcription factors (MRFs) are essential for skeletal myogenesis during embryonic development and for proper muscle regeneration [1-6]. *Myf5 *and *MyoD *are expressed in proliferating myoblasts, whereas *myogenin *and *MRF4 *are important in terminal differentiation [4,5,7,8]. In the absence of *MyoD*, muscle regeneration is impaired [9] possibly due to delayed differentiation of muscle precursor cells [5,7,10]. However, *MyoD *is also expressed in adult muscle fibres, albeit at low levels [11,12]. Conditions that damage muscle or change muscle phenotype often lead to changes of *MyoD *expression [13-15]. Nevertheless, when changes in *MyoD *expression occur, it is unclear how much is in fibres, myogenic cells or both [3,16]. Therefore, the activity and regulation of *MyoD *in normal muscle fibres is unknown.

The role of MyoD within muscle fibres is unknown. Differential expression of *MyoD *has been observed between muscles with distinct fibre type composition [11,17]. In several vertebrates, *MyoD *mRNA and protein is relatively more abundant in fast muscle and *myogenin *mRNA in slow muscle [12,17,18], suggesting a potential role in controlling muscle fibre phenotype. In the absence of MyoD, contractile function is perturbed due to diminished regulatory proteins within the muscle fibre [19]. However, *MyoD *is up-regulated in situations that cause the muscle fibres to change size. It has been proposed that MRFs are up-regulated to prevent muscle atrophy [11,20]. In fact, signalling pathways within muscle that reduce MyoD function are associated with muscle wasting [21]. Furthermore, denervation, a cause of rapid catabolism, has been shown to increase *MyoD *RNA and protein [22,23]. Interestingly, the use of a *myf5*-lacZ reporter demonstrated that *myf5 *within adult muscle fibres was increased by denervation [24]. This indicates that MRFs within adult muscle fibres may be controlled by the nerve and/or electrical activity and regulate some aspect of muscle function.

To date, *MyoD *regulation has been defined to occur through two elements. A 'core enhancer' around 20 kb 5' of the transcriptional start site drives early embryonic myoblast expression [25]. A bipartite element in the 5' proximal 6 kb contains a 'Distal Regulatory Region' (DRR) and a Proximal Regulatory Region (PRR), which together drive expression in adult muscle fibres and cultured muscle cells [17,26-29]. A transgenic construct, *MD6.0-lacZ*, in which the proximal 6 kb containing the DRR and PRR drives expression of nuclear-targeted β-galactosidase, mimics *MyoD *expression *in vivo*, showing appropriate preferential expression in some fast muscle fibres [12,17,27]. Deletion of the DRR element from the endogenous locus by homologous recombination leads to a reduction of *MyoD *expression in adult muscle [28], possibly from the fibre nuclei. Therefore, as endogenous *MyoD *is responsive to the nerve/electrical activity, we tested the hypothesis that elements in and around the DRR/PRR, which drives *MyoD *expression in fibre myonuclei, are also regulated by the nerve and/or electrical activity.

We used the *MD6.0-lacZ *reporter mouse to show that this regulatory element of *MyoD *is activated in fibres by innervation and muscle activity. Comparison of DRR/PRR reporter and endogenous *MyoD *expression shows that activity-dependent regulation of *MyoD *is more complex than previously supposed. Denervation induces an opposite response from the DRR/PRR compared to endogenous *MyoD*. Altered activity levels with an intact nerve, during firing pattern maturation, induces a similar response in the DRR/PRR element compared to the endogenous gene. Furthermore, we show that maintenance of *MyoD *expression through the DRR/PRR element is dependent on positive feedback by MyoD, providing strong evidence that MyoD protein is transcriptionally active within normal adult muscle fibres.

## Results

### *MD6.0-lacZ *is specifically expressed in nuclei of MyHC-positive cells

**Figure 1:**
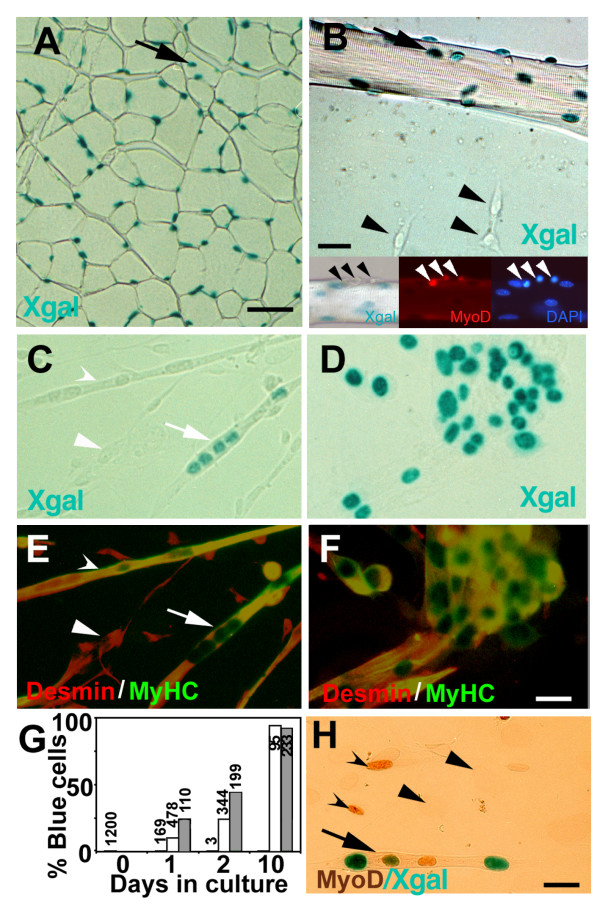
***MD6.0-lacZ *reporter is specifically associated with fibre nuclei**. **A**, Cross-section through the TA muscle of an *MD6.0-lacZ *adult transgenic mouse reacted with X-gal. Note the abundant and occasionally central (arrow) βgal^+ ^nuclei indicating that some reactive nuclei are within fibres. **B**, Single muscle fibre extracted from the EDL of an adult *MD6.0-lacZ *mouse, cultured for three days in growth medium and reacted with X-gal. The fibre (arrow) contains βgal^+ ^nuclei, whereas non-differentiated satellite cells coming from this fibre do not contain βgal (arrowheads). Insets: proliferating satellite cells on a cultured fibre express MyoD but not *MD6.0-lacZ*. MyoD in fibres is obscured by X-gal stain. **C-F**, Cell cultures from P0 *MD6.0-lacZ *limb muscle homogenates, grown for six days in growth medium and differentiated for one day (C, E) or 10 days (D, F). Cultures were reacted with X-gal (blue, C-F) and stained for desmin (red) to label myogenic cells, and for all MyHC (green) to label differentiated cells (E, F). Myonuclei weakly βgal^+ ^(concave arrowhead, stain visible only in E) or strongly βgal^+ ^(arrows) are in differentiated cells, whereas undifferentiated myogenic cells are βgal^- ^(arrowhead). After ten days differentiation, the majority of differentiated cells were strongly βgal^+^. **G**, MyHC expression precedes βgal accumulation as cultures differentiate. Desmin^+^/MyHC^- ^cells (black bars) lack βgal. Mononucleated and multinucleated MyHC^+ ^myocytes (white and grey bars, respectively) accumulate βgal with time in differentiation medium. Values above columns are the total numbers of cells observed in several dishes from three separate experiments. **H**, P0 *MD6.0-lacZ *culture after two days differentiation showing βgal (blue) only in a multinucleate cell, but MyoD (brown) in both myoblasts (concave arrowheads) and myotubes (arrow). Fibroblasts are unstained (arrowhead). Note that myotube nuclei unlabeled for βgal may be newly fused.

We investigated the expression pattern of the *MD6.0-lacZ *transgene in cross-sections from adult muscle and myoblast cultures. Four lines of evidence, taken together, indicate that the *MD6.0-lacZ *transgene is expressed postnatally in differentiated muscle fibres. First, the location and frequency of nuclear-targeted βgal in adult muscle cross-sections from *MD6.0-lacZ *mice show that many fibre nuclei express the transgene (Fig. 1A). Second, culture experiments using single fibres from dissociated *MD6.0-lacZ *muscles show βgal in fibre myonuclei, but not in mononucleate activated satellite cells on or migrating away from a single fibre, even though activated cells contain abundant MyoD protein (Fig. 1B). As reported previously, MyoD protein is very weakly detected in adult fibre nuclei, consistent with the greater sensitivity of βgal staining (Fig. 1B) [12]. Third, primary neonatal myoblast cultures with no differentiated MyHC-positive cells contain no βgal activity, although desmin-positive myogenic cells are present (Fig. 1G). Fourth, when such cultures are permitted to differentiate, βgal-containing nuclei appear following MyHC expression (Fig. 1C–G). After ten days differentiation, 92% of multinucleate myotubes contained βgal and MyHC as did 94% for mononucleated myocytes, showing that *MD6.0-lacZ *is activated upon myoblast differentiation (Fig. 1D–G). Together, these results indicate that the *MD6.0-lacZ *reporter is specifically expressed by fibre nuclei and not dividing myoblasts or activated satellite cells. *MD6.0-lacZ *acts, therefore, as a reporter of factors regulating *MyoD *expression within differentiated muscle.

### The DRR element of *MD6.0-lacZ *contributes to fibre expression

**Figure 2:**
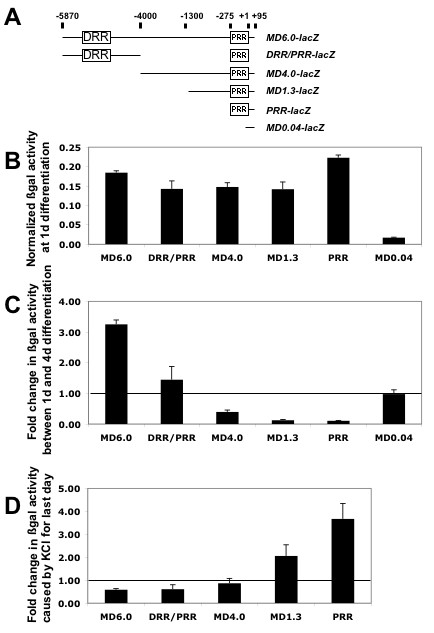
***MD6.0-lacZ *contains elements differentially regulated during differentiation**. Primary mouse myoblasts were transiently transfected with *MyoD-lacZ *reporter constructs and expression quantified relative to control *TK-luciferase*, as a control for transfection efficiency. Luciferase control plasmids showed no overall change in expression between 1 and 4 days. **A**, Schematic representation of the constructs used. **B**, MyoD reporter activity after 1 day in differentiating conditions. β-galactosidase activity in different MyoD reporter plasmids contained within the 6 kb region upstream of MyoD. **C**, The change in reporter activity at 4 days compared to 1 day in differentiating conditions. Expression from the DRR element increases during differentiation. **D**, The addition of KCl to reduce electrical activity during the final day of culture inhibits the changes in reporter activity seen during differentiation.

To determine the element(s) within *MD6.0-lacZ *that drive fibre expression, a series of deletion constructs (Fig. 2A) were transfected into primary myoblasts in cell culture and activity measured during differentiation into myotubes. All constructs containing more than the basal promoter express at similar levels in undifferentiated myoblasts and in cells triggered to differentiate overnight (Fig. 2B). After four days differentiation, however, the full length *MD6.0-lacZ *construct shows significant up-regulation (Fig. 2C), with 72% of cells having detectable X-gal reactivity (data not shown). Removal of the DRR by truncation to 4 kb completely abolishes the up-regulation, and further truncations containing only the PRR element lead to a repression of activity during differentiation, although some residual activity does remain (Fig. 2C). The ratio of *PRRlacZ *activity to luciferase control plasmid was 0.3 ± 0.001 and 0.001 ± 0.003 after 1d and 4d differentiation, respectively. In contrast, the low activity of the empty PD46lacZ control vector did not change during differentiation. These data suggest that the PRR becomes repressed during myotube maturation, rather than simply failing to respond to signals that increase expression during maturation. Furthermore, the repressive effect of the PRR could be reversed by addition of the DRR, showing that the DRR contains an element activated during differentiation (Fig. 2C). Nevertheless, the overall activity of the *MD6.0-lacZ *construct is significantly higher than that of the DRR/PRR alone, indicating that other aspects of the *MD6.0-lacZ *sequence are essential for full activity (Fig. 2C). We conclude that the activity of multiple elements within the *MD6.0-lacZ *construct change during muscle differentiation, with those in and around the DRR being activated and those in the PRR being repressed.

### *MD6.0-lacZ *is induced in fibres as neonatal mice mature

**Figure 3:**
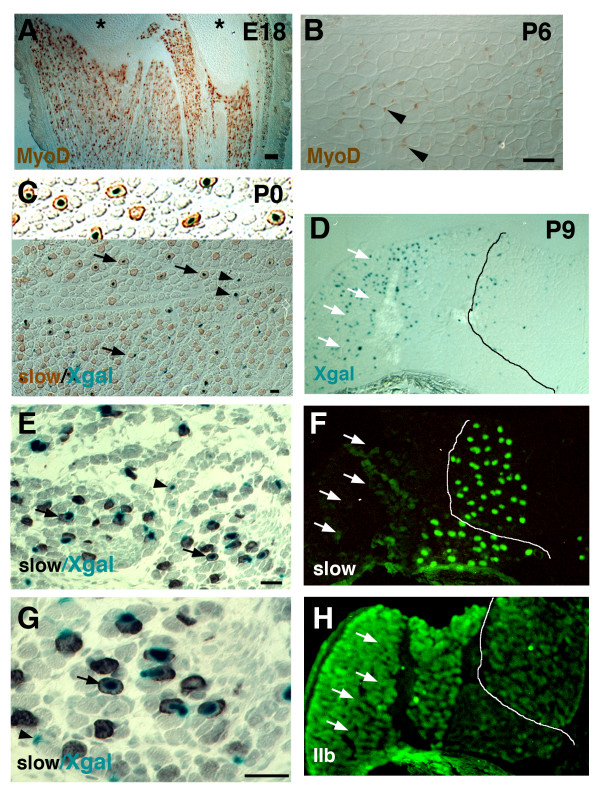
***MD6.0-lacZ *is active in slow and fast fibre types as each matures**. Muscle cryosections from E18 (A) and P6 (B) showing MyoD protein, or P0 (C, E, G) and P9 (D, F, H) *MD6.0-lacZ *mice reacted with X-gal. Scale bar = 50 μm. **A, B**, Late embryonic (A, E18, longitudinal section) and P6 (B, transverse section) lower hindlimb muscle showing the decline in MyoD (brown) in fibre nuclei and presence in myogenic cells (arrowheads). Asterisks, cartilage. **C**, EDL/TA section stained for slow MyHC (brown) and X-gal (blue). Note correlation blue nuclei in slow fibres (arrows; brown), magnified in inset at top. Blue nuclei are more rarely associated with fast fibres (arrowheads). **E, G**, Plantaris and lateral gastrocnemius muscles stained for slow MyHC (black). At P0, the majority of βgal^+ ^(blue) fibres express slow MyHC (arrows), although rare βgal^+ ^associate with fast fibres (arrowheads). **D, F, H**, By P9, serial cross-sections of anterolateral region reacted for X-gal (D), slow (F) and fast IIb MyHC (H) show the majority of βgal^+ ^fibres are within a fast IIb muscle area of TA (arrows) and little remains within muscle containing slow fibres. Line marks border of TA (left) and EDL (right).

In contrast to *MD6.0-lacZ*, endogenous MyoD protein is expressed in myoblasts in both myoblast cultures and embryonic hindlimb muscle, as well as in muscle fibres (Figs 1H and 3A). Thus, different regulatory elements within the *MyoD *gene regulate myoblast and myofibre expression. After birth, MyoD is down-regulated, becoming less abundant in myoblasts and barely detectable in most fibre nuclei at postnatal day 6 (P6; Fig. 3B). As with endogenous MyoD, βgal activity from *MD6.0-lacZ *is detected in only a few fibres in neonates (Fig. 3C,E,G) [27]. Interestingly, at this stage, βgal activity is preferentially localised within central nuclei of large slow fibres (Fig. 3C,E,G). This preferential slow fibre localisation is found in all hindlimb muscles analysed. In postnatal day 0 (P0) tibialis anterior (TA), 44% (11/25) of slow fibres and 5% (9/187) of fast fibres react for X-gal. Similarly, in P0 lateral gastrocnemius, 24% (10/41) of slow fibres but only ~1% (1/73) of non-slow fibres contained βgal activity. Therefore, reporter expression is restricted to the more mature fibres at this stage, many of which are slow fibres.

After P7, low levels of MyoD become detectable in some fast fibre nuclei [12]. As muscles mature, the expression of *MD6.0-lacZ *is not maintained in slow fibres but instead accumulates in muscle regions rich in fast fibres (Fig. 3D,F,H). This pattern is similar to that previously described in adult *MD6.0-lacZ *muscles and to the pattern of endogenous MyoD protein expression in maturing rodent muscle [12]. Thus, commencing just before P9, *MD6.0-lacZ *reporter expression up-regulates preferentially within fast fibres in regions where muscle fibres will subsequently be predominantly large type IIb fibres. This up-regulation parallels maturation of neural firing patterns [30], raising the possibility that nerve-dependent muscle activity regulates the DRR/PRR element.

### *MD6.0-lacZ *expression is maintained by activity

**Figure 4:**
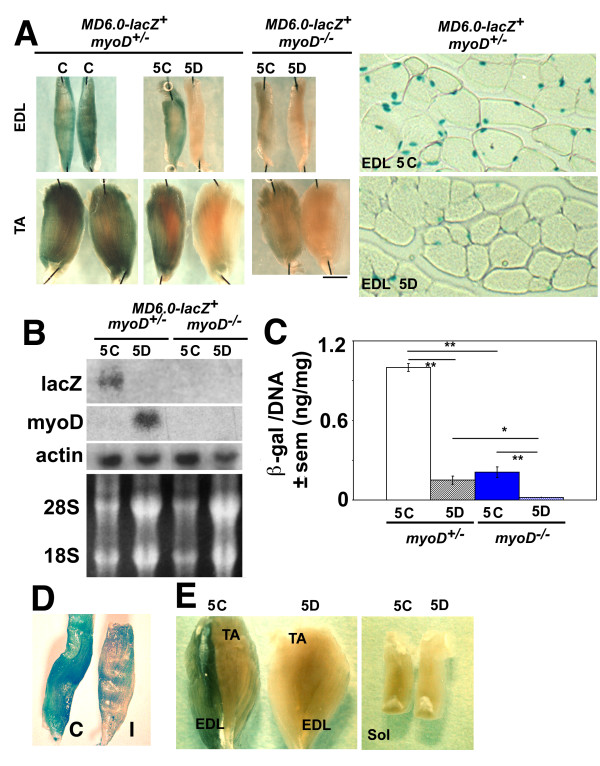
**Innervation, activity and *MyoD *are required for *MD6.0-lacZ *activity**. Analysis of EDL, TA and soleus (Sol) muscles from control (C), 5 days denervated (5D) and contralateral control (5C) adult *MD6.0-lacZ*. *MyoD*^+/- ^or *MyoD*^-/- ^mice. **A**, Whole muscles and muscle cross-sections reacted for X-gal. X-gal staining is reduced following 5 days denervation in both *MyoD*^+/- ^and *MyoD*^-/-^. Loss of *MyoD *reduces reporter activity. **B**, Northern analysis of total RNA isolated from TA/EDL muscles. Endogenous *MyoD *expression is increased following denervation whereas *lacZ *expression is decreased. Actin expression is unchanged upon denervation but 28S and 18S rRNA transcripts are up-regulated. *MyoD *is required to maintain *lacZ *mRNA levels. **C**, β-galactosidase activity relative to DNA content within TA/EDL homogenates. Asterisks indicate significant difference * P < 0.01, ** P < 0.001. **D**, EDL muscles from 5 day immobilised leg (I) and contralateral control leg (C) of *MD6.0-lacZ *adult mouse after X-gal staining. **E**, Whole muscles from a *MD6.0-lacZ *mouse reacted for X-gal five days after unilateral sciatic denervation. As in fast muscles, soleus staining is reduced following denervation. Staining is less in innervated soleus compared to TA/EDL. Note that the slower medial surface of TA contains less βgal than either superficial TA or EDL.

**Figure 5:**
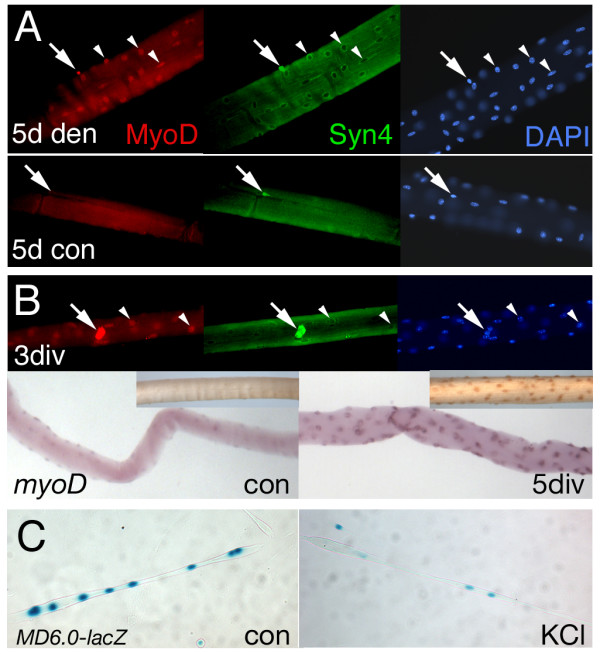
***MD6.0-lacZ *reporter and endogenous *MyoD *differ in response to activity**. **A**, Immunoreactivity of MyoD (red) and Syndecan-4 (green) in single fibres isolated from five day denervated or contralateral control EDL muscles. Note rise in MyoD in the small nuclei of Syndecan-4^+ ^satellite cells (arrows) after denervation. Large fibre nuclei (arrowheads) also have more MyoD after denervation. **B**, Expression of MyoD protein in activated satellite cell (arrow) and fibre (arrowhead) nuclei after three days culture *in vitro *(upper panels). In situ hybridisation for *MyoD *mRNA (lower panels; and protein, inset) in fibres from an innervated EDL muscle immediately after isolation (con) or after five days floating culture *in vitro *(5 div) in a well without Matrigel coating. **C**, Myotubes derived from TA/EDL of P0 *MD6.0-lacZ *mice were allowed to mature in differentiation medium for 11 days (con) or treated for the final four days with medium supplemented with 10 mM KCl (KCl) to induce depolarization. Cultures were analysed by X-gal staining (blue).

To examine the effect of innervation on *MyoD *expression, mouse lower hindlimb muscle was denervated by unilateral sciatic nerve section. At 5 days post-operation, expression of the *MD6.0-lacZ *reporter declines at both the protein and mRNA levels. Wholemount X-gal stain reveals a striking loss of reaction (Fig. 4A). Contralateral and mock-operated muscles show no significant change in expression (Fig. 4A and data not shown). The fold decrease elicited by denervation in *lacZ *mRNA is 4 ± 1 (n = 5) for *lacZ*/*actin *mRNA level and possibly slightly greater for βgal protein at 7 ± 2 (n = 3) for βgal content/total DNA (Fig. 4B,C). At five days post-operation no decline in muscle wet mass is apparent, but ribosomal RNA is significantly increased relative to contralateral control muscles, whereas actin mRNA is not. A similar loss of reporter activity is observed when *MD6.0-lacZ *lower hindlimb muscles are immobilised for five days compared to contralateral control muscles (Fig. 4D). Therefore, activity maintains *MD6.0-lacZ *reporter expression within the muscle fibre. As reported previously [12], *MD6.0-lacZ *reporter activity and endogenous *MyoD *expression is less in innervated slow soleus muscle than in innervated fast EDL muscle. This low level of reporter activity in soleus is also nerve-dependent (Fig. 4E). Thus, slow and fast nerves each up-regulate *MyoD *reporter activity in their respective target muscles. The data suggest that the up-regulation of endogenous *MyoD *in fibres induced by maturing innervation and/or activity is mediated by elements within the *MD6.0-lacZ *transgene. To test further the role of activity in *MD6.0-lacZ *expression we examined myotubes treated with high K^+ ^medium, which has previously been shown to down-regulate *MyoD *expression [31]. Myotubes were generated *in vitro *from bulk neonatal *MD6.0-lacZ *hindlimb myoblast cultures and from satellite cells derived from single fibre culture. In both cases, treatment of myotubes with KCl led to a decline in both βgal accumulation and detectable MyoD protein (Fig. 5C and data not shown). Similarly, KCl treatment of primary myotubes transiently transfected with *MD6.0-lacZ *reduces βgal activity (Fig. 2D). These results argue that the K^+^-induced suppression of *MyoD *expression in nascent muscle fibres is mediated through the *MD6.0-lacZ *element.

### Activity has opposing effects on DRR and PRR elements

To examine the mechanism of activity-dependent regulation of the *MyoD *gene through the DRR and PRR elements within *MD6.0-lacZ *in more detail we compared the effect of reducing electrical activity on *lacZ *reporter expression from a series of deletion constructs. Application of 10 mM KCl to primary myotubes completely stops the spontaneous twitching that commences three days after differentiation (data not shown). Expression from constructs containing the DRR are reduced by KCl. In contrast, expression from the PRR element is increased (Fig. 2D). Electrical activity in immature fibres appears, therefore, to enhance *MD6.0-lacZ *expression through the DRR element by over-riding an activity-dependent inhibitory signal acting on the PRR element. Taken together with our findings on denervated muscle, these results show that electrical activity acts on multiple regulatory elements within the *MyoD *gene, at least two of which lie within the *MD6.0-lacZ *construct.

### Activity-dependent suppression of endogenous *MyoD *expression acts through elements outside *MD6.0-lacZ *construct

As reported previously for whole lower hindlimb [11,16], and contrary to the behaviour of the *MD6.0-lacZ *reporter, endogenous *MyoD *mRNA is increased at five days post-denervation (Fig. 4B). The ratio of *MyoD *mRNA to *actin *mRNA is elevated approximately sevenfold (from a longer exposure, data not shown). The decline in *lacZ *mRNA suggests that nerve activity maintains a basal level of *MyoD *expression in certain muscle fibres through the DRR/PRR enhancer, but that the loss of this expression on denervation is over-shadowed by up-regulation of endogenous *MyoD *driven through other elements outside the *MD6.0-lacZ *transgene.

Satellite cells become activated upon denervation [32,33], which would be expected to increase *MyoD *mRNA without raising *MD6.0-lacZ *expression (Fig. 1). However, the large increase in endogenous *MyoD *mRNA after denervation, together with immunohistological analyses [3,34], suggested that much of the *MyoD *mRNA expressed after denervation is in fibres themselves. To distinguish the contribution of these two processes, we first examined MyoD protein and mRNA changes in cultured single fibres, which do not twitch *in vitro*, showing that electrical activity is essentially eliminated. *MyoD *mRNA expression was weakly detectable around nuclei in acutely-isolated fast fibres from un-manipulated adult mice, but increased substantially after culture of fibres for five days *in vitro *(Fig. 5B). Moreover, immunological detection of MyoD protein either three or five days after explant gave a similar result, with increased MyoD immunoreaction in both fibre nuclei and in satellite cell-derived nuclei, which we identified by their strong Syndecan-4 expression (Fig. 5B). Conversely, Xgal reaction decreased within the isolated fibres with time in culture (data not shown). Thus, the normal in vivo environment suppresses endogenous *MyoD *mRNA and protein within fibres.

To determine whether electrical activity itself suppresses MyoD accumulation, we denervated adult muscles *in vivo*, waited five days and then isolated single fibres and detected MyoD by immunofluorescence (Fig. 5A). MyoD protein was detected within many nuclei of fibres from denervated muscle, but was essentially undetectable in fibres from contralateral innervated muscle. In addition, MyoD was strongly up-regulated in the morphologically-distinct nuclei of Syndecan-4^+ ^satellite cells. Taken together with the strong up-regulation of *MyoD *mRNA and loss of *lacZ *mRNA in Northern blots of denervated muscle (Fig. 4B), these data show that the up-regulation of MyoD in fibres requires elements outside the *MD6.0-lacZ *construct.

### *MD6.0-lacZ* is down-regulated in *MyoD*^-/- ^fibres

In myoblasts, MyoD can positively auto-regulate its own expression [26,35]. Its action in adult fibres is unknown. We therefore asked whether *MyoD *expression in fibres *in vivo *is regulated by endogenous MyoD. Expression of the fibre specific reporter, *MD6.0-lacZ *was examined in a *MyoD *null mutant background. Strikingly, *lacZ *mRNA is substantially lower in TA/EDL muscle of *MD6.0-lacZ*; *MyoD*^-/- ^mice compared to controls (Fig. 4B). Similarly, βgal staining within TA and EDL muscles of *MyoD*^-/- ^mice is strongly decreased compared to that within *MyoD*^+/- ^mouse muscles (Fig. 4A). Furthermore, βgal activity is decreased 5-fold in TA/EDL homogenates of *MyoD*^-/- ^muscles compared to levels within *MyoD*^+/- ^muscles (Fig. 4C). These results demonstrate that in the absence of *MyoD*, the *MD6.0-lacZ *reporter is down-regulated. Thus, MyoD protein acts positively to maintain *MD6.0-lacZ *reporter expression in innervated adult muscle fibres.

The decline in *MD6.0-lacZ *expression of *MyoD*^+/- ^mice after denervation is of a similar magnitude to the difference in *MD6.0-lacZ *expression between *MyoD*^+/- ^and *MyoD*^-/-^. It was therefore conceivable that a decline in activity of endogenous MyoD following denervation (despite its increased abundance) might account for the loss of reporter expression. To test this hypothesis, *MD6.0-lacZ*; *MyoD*^-/- ^mice were denervated. Following denervation of *MyoD*^-/- ^muscle, the *MD6.0-lacZ *reporter is further down-regulated. X-gal staining on EDL and TA muscles from five day denervated *MD6.0-lacZ*; *MyoD*^-/- ^mice shows a decrease in βgal activity to undetectable levels compared to contralateral control legs (Fig. 4A). Quantification of βgal within *MyoD*-deficient muscles shows a significant decrease after five days denervation (Fig. 4C). This decrease mirrors the decline of *lacZ *mRNA to essentially undetectable levels (Fig. 4B). These results demonstrate that the down-regulation of *MD6.0-lacZ *reporter upon denervation is independent of MyoD positive feedback. Thus, the DRR/PRR enhancer region of *MyoD *is independently regulated by both innervation and endogenous MyoD.

## Discussion

The data presented provide strong evidence that MyoD protein is present and active within many adult fast muscle fibres. This *MyoD *expression is regulated by innervation and muscle activity, which act both positively and negatively on several separable regulatory elements in the *MyoD *gene to fine tune *MyoD *expression. The data implicate MyoD in the adaptation of muscle to altered physiological activity.

Cultured myoblasts and activated satellite cells do not express *MD6.0-lacZ*. On terminal differentiation, most myotubes in cell culture activate *MD6.0-lacZ*. In mice, *MD6.0-lacZ *expression is only observed in fibres. These data are consistent with the late onset of *MD6.0-lacZ *expression in the embryo, the correlation of differentiation failure with loss of *MD6.0-lacZ *expression and the requirement for the DRR element for *MyoD *expression in adult muscle [28,36,37]. Thus, myoblast *MyoD *expression is not driven through the *MD6.0-lacZ *element, at least after birth. DRR-driven reporters do express at low levels in proliferating myoblasts in culture [26,29](Fig. 2). However, our data show that such expression may not be significant in the *in vivo *context, with the possible exception of satellite cell activation in regenerating muscle [29]. In our hands, essentially all cultured mononucleated cells expressing *MD6.0-lacZ *also contain the terminal differentiation marker MyHC. Moreover, denervation or fibre explant induce MyoD accumulation in satellite cells without up-regulating *MD6.0-lacZ*. Taken together with the differential expression of both *MyoD *and *MD6.0-lacZ *between adult muscles of distinct contractile character [11,12,17,27], these data provide compelling evidence that most, probably all, *MD6.0-lacZ *expression is in differentiated muscle fibres. However, not all muscle fibres express the *MD6.0-lacZ *reporter simultaneously.

Altered nerve activity appears to drive postnatal *MyoD *and *MD6.0-lacZ *expression changes. Innervation is required for muscle maturation. For example, firing pattern determines mature fast fibre types [38]. As fast fibres and their innervation mature during the first postnatal week, *MD6.0-lacZ *expression emerges predominantly in the fastest fibres of fast muscles, which have burst firing but low overall electrical activity levels [30,39]. Subsequently, fast *MD6.0-lacZ *reporter expression is nerve-dependent. We suggest, therefore, that maturing fast firing promotes *MD6.0-lacZ *expression in fast fibres. Conversely, prior to fast fibre maturation, *MD6.0-lacZ *expression is confined to small numbers of mainly large slow fibres. This expression declines as slow fibres mature further. Firing patterns in newborn mice are unknown, but by P12 rat slow soleus motor units have a mature electrical firing rate [30], suggesting the maturing slow firing pattern suppresses expression of *MD6.0-lacZ *in slow fibres before the second postnatal week. Nevertheless, adult slow soleus fibres require innervation to maintain their low levels of *MD6.0-lacZ *expression. Even a reduction in activity through leg immobilisation leads to decline in *MyoD *reporter expression, indicating that it is the activity elicited in muscle by the nerve that is required, rather than other 'trophic' factors. Thus, the *MD6.0-lacZ *reporter appears to contain elements that integrate electrical activity-dependent signals in distinct fibre types during development and maturation.

Denervation of adult muscle leads to a decrease in *MD6.0-lacZ *activity. Therefore, in the adult fibre, elements inside *MD6.0-lacZ*, possibly the DRR, integrate activity-dependent signals. Unlike the *MD6.0-lacZ *reporter, various MRF mRNAs are induced by denervation, although because satellite cells become activated the location of the up-regulated mRNAs has been unclear [11,16,32,33,35,40,41]. Transient up-regulation of MyoD protein early after denervation has been reported in rat satellite cells and muscle fibres [3]. We confirm up-regulation of MyoD in both fibre and satellite cell nuclei on denervation. Similarly, a *myf5 *reporter is induced in fibre nuclei after denervation. However, *myf5 *is not expressed in innervated fibres [24], which indicates that *myf5 *and *MyoD *are regulated differently in adult muscle fibres.

Our data show that maintenance of normal levels of *MD6.0-lacZ *expression in adult fast fibres is dependent upon MyoD itself. MyoD is well known to regulate its own expression during myogenic conversion of various cell types *in vitro *[42]. The action of MyoD in fibres is probably a cell autonomous positive auto-regulatory loop as MyoD mRNA and protein are differentially accumulated in adult fast fibres, just like *MD6.0-lacZ *[11,12,17]. The DRR is required in the endogenous *MyoD *gene for normal adult expression, making it an obvious candidate site for MyoD positive feedback [28]. We show that this region is positively regulated by activity during myotube maturation. However, the DRR, PRR and intervening elements each contain several potential MRF binding sites. In addition, a MEF2 site in the DRR helps drive myotube expression [43]. Our data do not preclude cooperative roles for other factors, such as Mef2, in regulating MyoD in fibres. As MyoD can collaborate with Mef2 to enhance transcription in the absence of a MyoD binding site [44], MyoD could directly enhance its own expression without DNA binding. Further work will be required to determine where within the *MD6.0-lacZ *region MyoD actually binds in mature fibres. To conclude, we have thus identified two factors, MyoD protein and fibre activity, that are required for essentially all expression of *MD6.0-lacZ *in adult fibres.

**Figure 6:**
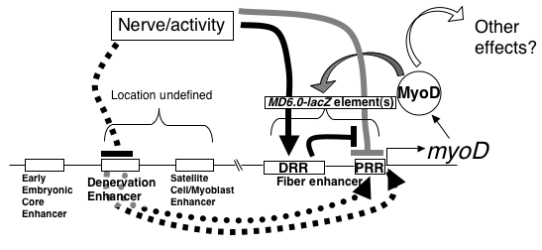
**Model of *MyoD *regulation in adult muscle fibres**. *MD6.0-lacZ *element(s) constituting a 'Fibre Enhancer' integrate two signals, positive auto-regulation by MyoD and nerve/activity-dependence, and target them onto the native *MyoD *promoter. Activity effects on the DRR/PRR region comprise suppression of PRR (grey line) and activation of DRR, which predominates in the intact DRR/PRR region (black lines). Another activity-dependent 'Denervation Enhancer' mediates the activation of *MyoD *in adult fibres after denervation by one of two routes. Activity may suppress this enhancer, which otherwise directly activates the basal promoter (dashed lines). Alternatively, this enhancer may co-operate with the PRR (dotted arrow) to activate the basal promoter when direct effects of nerve on the DRR/PRR region (solid lines) are absent. In either case, upon denervation the poised low-level *MyoD *expression driven through *MD6.0-lacZ *Fibre Enhancer is lost, but MyoD rises driven by the Denervation Enhancer. Despite the increased level of nuclear MyoD protein, lack of nerve/activity prevents maximal activation of the *MD6.0-lacZ *transgene. In innervated fibres, we propose that subtle differences in activity in otherwise similar fibres lead to changes in *MyoD *expression that are amplified by positive feedback (grey arrow) through the Fibre Enhancer, thereby explaining fibre-to-fibre variation in *MD6.0-lacZ *and *MyoD *expression. Active MyoD in these fibres may mediate some effects of the nerve, such as the rate of anabolism (open arrow). Elsewhere in the gene, elements missing from *MD6.0-lacZ *control expression in, for example, early embryonic myogenic cells and other myoblasts.

A separate mechanism requiring elements outside *MD6.0-lacZ *up-regulates *MyoD *after denervation (Fig. 6). Suppression of activity in culture, or denervation *in vivo*, down-regulates *MD6.0-lacZ*, probably through loss of the positive effect on the DRR region. In contrast, denervation up-regulates expression of the intact *MyoD *gene in fibres. The ability of activity to suppress expression from constructs containing the PRR but lacking the DRR, suggests that elements outside *MD6.0-lacZ *region may interact with the PRR. In our view, the simplest resolution of these data is to suggest that a general effect of innervation is to suppress *MyoD *through elements outside the *MD6.0-lacZ *construct. However, particular kinds of activity can overcome this suppression, perhaps by acting positively through the DRR. For example, the parallel increase in *MD6.0-lacZ *activity and MyoD during fast fibre maturation may act in this manner. These findings indicate that denervated fibres have a unique status, at least in terms of MRF expression, and do not appear to return to an 'immature' myotube-like state. In summary, activity-dependent regulation of *MyoD*, as well as embryonic and myoblast expression, requires elements outside the DRR/PRR.

The effects of electrical activity appear complex and dispersed in the *MyoD *locus. Whereas nascent cultured myotubes, and early embryonic fibres *in vivo *[36], express *MD6.0-lacZ *highly when they are spontaneously active, *MD6.0-lacZ *is not highly expressed in fibres at birth, when innervation is present but firing pattern is immature. This indicates that certain kinds of activity may suppress *MD6.0-lacZ*. Consistent with this, in the absence of the DRR, activity inhibits expression from the PRR element in cultured myotubes. However, neonatal denervation does not lead to *MD6.0-lacZ *up-regulation (A. Brack, unpublished observation). Thus, it appears that positive effects of particular kinds of activity acting through the DRR can override the suppressive effects acting via the PRR.

In both *MyoD*^+/- ^and *MyoD*^-/- ^mice, *MD6.0-lacZ *is differentially expressed between fast and slow muscle and expression declines following five days denervation. Therefore, MyoD is not needed for innervation to promote differential reporter expression between fibre types. The parallel decline of *MD6.0-lacZ *mRNA and βgal protein after denervation and in *MyoD*^-/- ^mice show that changes in reporter protein turnover do not account for all changes in reporter activity [45,46]. Thus, multiple regulatory mechanisms co-ordinate the expression of *MyoD *in adult muscle tissue and those acting within *MD6.0-lacZ *mediate nerve- and MyoD-dependent activation in fibres in an apparently mutually independent manner (Fig. 6). The positive feedback of MyoD on the DRR/PRR appears to sensitize innervated fibres to changes in activity. Based on the ability of activity to promote transcription through the DRR in cultured cells, we suggest that the positive effects of electrical activity on *MD6.0-lacZ in vivo *act through the DRR, which is required for normal adult *MyoD *expression [28].

The role of MyoD is not completely understood, but the function of MyoD in adult fast fibres is unlikely to be restricted to auto-regulation (see below). To date, the mild fibre phenotype of *MyoD *null mice has shed insufficient light on the role of MyoD in fibres [12,47-49]. Nevertheless, our findings show that MyoD is present and transcriptionally-active in large numbers of muscle fibres in adult mice.

MyoD has been proposed to limit muscle atrophy [11,20]. Muscle growth can occur by both the fusion of myoblasts from activated satellite cells [50] and by the anabolic accumulation of cytoplasm in an existing fibre [51]. MyoD is present in both satellite cells and fibre nuclei where, as we show, they are controlled by different enhancer elements. It is tempting to speculate that *MyoD *could have a role in both forms of muscle growth, with the DRR/PRR region involved in anabolic cytoplasmic growth (Fig. 6). MyoD may control developmental MyHCs in nascent fibres [52]. After maturation, fibre MyoD expression is primarily associated with the larger fast fibre types [12,17]. We note that *MD6.0-lacZ *is not always expressed in the largest fibres of a particular type. Could reporter expression reflect fibres in a particular anabolic state e.g. in the process of increasing their size? In the *MyoD *null mouse there is a shift of fast fibres to a slower phenotype [12] and during hindlimb unloading the *MyoD *null mouse fails to up-regulate *myHC IIb *gene expression [48]. Conversely, over-expression of *MyoD *activates the promoter of the *myHC IIb*, but not the promoters of *myHC IIx *or *myHC IIa *gene [53]. This suggests that MyoD has a functional role in controlling the IIb myosin gene and therefore muscle fibre phenotype. The present results suggest the DRR/PRR region, by controlling fibre expression of *MyoD*, has a significant role in determining the physiological phenotype of adult skeletal muscle.

## Conclusion

Our data show that *MyoD *regulation is more complex than previously supposed. Two factors, MyoD protein itself and fibre activity are required for essentially all expression of the 6 kb proximal enhancer/promoter (DRR/PRR) of *MyoD *in adult fibres. We propose that modulation of MyoD positive feedback by electrical activity determines the set point of *MyoD *expression in innervated fibres through the DRR/PRR element.

## Methods

### Mouse rearing and procedures

Mice were fed *ad libitum *in plastic cages with wire mesh lids on a 12 h light/dark cycle. *MD6.0-lacZ *mice, generously provided by Dr S.J. Tapscott, were bred to *MyoD*^-/- ^[54](Rudnicki *et al*., 1992). Adult animals were between 4 and 18 months old. Sex-matched littermates were controls. *MD6.0-lacZ *transgene dosage was constant within all experiments. Animals were anaesthetized by successive intraperitoneal injections of Xylazine HCl (20 μg/g body weight) and ketamine HCl (100 μ/g body weight). Denervation was by sciatic nerve section at the mid thigh level. Immobilisation was by applying a plaster cast to lower leg and foot. Animals were killed by CO_2 _inhalation followed by cervical dislocation. All experiments were performed under Home Office licence after local ethical review.

### Histology and Immunocytology

Fibre types were identified immunohistochemically or immunofluorescently in unfixed cryosectioned muscle for IIb MyHC (BF-F3), IIa MyHC (A4.74), slow β-cardiac MyHC (A4.840) and slow and IIa MyHC (N2.261) [55,56]. MyoD in sections was detected with a polyclonal antiserum kindly provided by A. John Harris as described [12]. X-gal reaction was performed on paraformaldehyde fixed muscles, cryosections were restained overnight. Single Extensor Digitorum Longus (EDL) fibres were dissociated with collagenase as described [56], fixed in 4% paraformaldehyde within 2 hrs, permeabilized in 0.3% Triton-X100, incubated with Syndecan-4 [57] and MyoD (1/200, clone 5.8A, BD Pharmingen) antibodies, reacted with Alexa488-conjugated goat anti-chicken IgG (1/200, Molecular probes) and rhodamine-conjugated donkey anti-mouse IgG (1/200, Chemicon). Nuclei were stained with DAPI prior mounting.

### DNA and protein extraction and β-galactosidase assay

Muscle was weighed, homogenised in Galacto-Light lysis buffer with 0.2 mM PMSF and 5 μg/ml leupeptin, centrifuged to remove insoluble material and stored frozen. Aliquots were analysed for protein (BCA kit, Pierce, IL, USA), DNA by addition of Hoechst 33258 [58] and βgal activity by Galacto-Light kit (Tropix Inc, MA, USA).

### RNA analysis

Total RNA was extracted from *Tibialis Anterior *(TA) and EDL muscles [59]. Total RNA from each muscle was run to ensure proper comparison of RNA levels after denervation, blotted and probed with [^32^P] random-primed cDNA probes to *MyoD*, *lacZ *and human β-*actin *cDNA (which readily cross-reacts with mouse α-actin) as a loading control. In situ mRNA hybridisation [60] was performed with digoxigenin-labelled riboprobes on single EDL fibres.

### Cell Culture

Single EDL fibres dissociated with collagenase were cultured and analyzed as described [56]. Neonatal hindlimb muscle cells were plated at 20 cells/mm^2 ^[61]. MyoD in cultured cells was detected with a polyclonal antiserum kindly provided by A. John Harris as described [62]. For transfection, primary myoblasts were obtained as previously described [63] and cultured in growth medium (Ham's F10, 20% foetal bovine serum, 2.5 ng/ml bFGF) on ECM (Sigma) coated dishes. Differentiation was induced by switching to differentiation media (Dulbecco's modified Eagle medium supplemented with 4% horse serum).

### Cell Transfection

To assess MyoD promoter activity, myoblasts were co-transfected using lipofectamine 2000 (Invitrogen, CA) with 2 μg DNA of β-galactosidase *myoD *reporters as described previously [27] and a pGL2-TK-Luciferase plasmid to normalize for transfection efficiency. After transfection cells were washed and left overnight in Growth medium, transferred to differentiation medium, which was replaced daily for between 1 and 4 days. To assess the role of electrical activity on myotubes, 10 mM KCl was added after 3 days differentiation for 24 hours. Cells were lysed and reporter activity quantified using Galacto-light assay (Tropix) and Dual light luminometer (Turner Biosystems).

## List of Abbreviations

MRF myogenic regulatory factor

DRR Distal regulatory region

PRR Proximal regulatory region

MyHC Myosin heavy chain

## Authors' contributions

SBC analysed the mice. ASB performed the cell culture experiments. SAB performed the denervation Northern. SBC and ASB helped design the study and write the manuscript. SMH obtained the money, designed the study and wrote the manuscript. All authors read and approved the final manuscript.
